# Workplace mental health: developing an integrated intervention approach

**DOI:** 10.1186/1471-244X-14-131

**Published:** 2014-05-09

**Authors:** Anthony D LaMontagne, Angela Martin, Kathryn M Page, Nicola J Reavley, Andrew J Noblet, Allison J Milner, Tessa Keegel, Peter M Smith

**Affiliations:** 1 Population Health Strategic Research Centre, School of Health & Social Development, Deakin University, Burwood, VIC, Australia; 2 Melbourne School of Population and Global Health, University of Melbourne, Melbourne, VIC, Australia; 3 The Tasmanian School of Business and Economics, University of Tasmania, Hobart, TAS, Australia; 4 Deakin Graduate School of Business, Deakin University, Burwood, VIC, Australia; 5 Centre for Ergonomics and Human Factors, Department of Human Biosciences and Public Health, Latrobe University, Bundoora, VIC, Australia; 6 Department of Epidemiology & Preventive Medicine, Monash University, Melbourne, VIC, Australia; 7 Institute for Work & Health, Toronto, ON, Canada; 8 Dalla Lana School of Public Health, University of Toronto, Toronto, ON, Canada

## Abstract

**Background:**

Mental health problems are prevalent and costly in working populations. Workplace interventions to address common mental health problems have evolved relatively independently along three main threads or disciplinary traditions: medicine, public health, and psychology. In this *Debate* piece, we argue that these three threads need to be integrated to optimise the prevention of mental health problems in working populations.

**Discussion:**

To realise the greatest population mental health benefits, workplace mental health intervention needs to comprehensively 1) protect mental health by reducing work–related risk factors for mental health problems; 2) promote mental health by developing the positive aspects of work as well as worker strengths and positive capacities; and 3) address mental health problems among working people regardless of cause. We outline the evidence supporting such an integrated intervention approach and consider the research agenda and policy developments needed to move towards this goal, and propose the notion of integrated workplace mental health literacy.

**Summary:**

An integrated approach to workplace mental health combines the strengths of medicine, public health, and psychology, and has the potential to optimise both the prevention and management of mental health problems in the workplace.

## Background

Mental health problems are common in the working population, and represent a growing concern, with potential impacts on workers (e.g., discrimination), organisations (e.g., lost productivity), workplace health and compensation authorities (e.g., rising job stress-related claims), and social welfare systems (e.g., rising working age disability pensions for mental disorders) [1]. Growing awareness of this issue has been paralleled by the rapid expansion of workplace interventions to address common mental health problems in the workplace setting, particularly as a means to prevent, detect, and effectively manage depression and anxiety [2-4].

**Figure 1:**
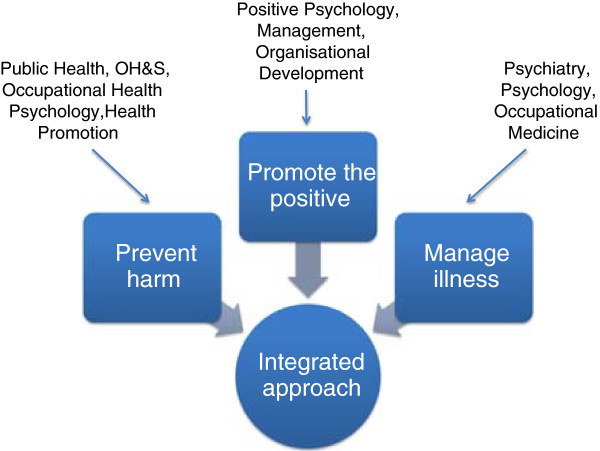
The three threads of the integrated approach to workplace mental health.

Workplace interventions to address common mental health problems have evolved relatively independently along three main threads or disciplinary traditions: medicine, public health, and psychology (Figure 1). In this *Debate* piece, we present two premises relating to 1) the high prevalence of such problems and disorders in the working population and 2) that working conditions are a major modifiable risk factor, then argue that the three intervention traditions or threads need to be integrated to achieve the greatest population mental health benefits. An integrated approach would 1) protect mental health by reducing work–related risk factors; 2) promote mental health by developing the positive aspects of work as well as worker strengths and positive capacities; and 3) address mental health problems among working people regardless of cause. Our aim in presenting this framework is to support the achievement of best practice in workplace mental health for the full range of relevant stakeholders: workers, employers, industry groups, labour organisations, policy-makers, health professionals, researchers, and others.

### Premise One: mental health problems are prevalent in working populations

Mental health problems, both clinical (e.g., major depression, anxiety disorders) and sub-clinical (e.g., psychological distress), are very common in working populations. This *Debate* piece focuses on the workplace setting - and thus the *working* population. However, it is important to acknowledge the complementary need for a more comprehensive view of the entire *working-age* population, which includes the unemployed, and those not in the labour force due to disability or other reasons [5]. Given growing labour market flexibility and rising levels of unemployment and underemployment in many Organisation for Economic Cooperation & Development (OECD) countries [6], addressing worklessness as well as work is now particularly important. In a recent review, the OECD estimated that similar proportions of the industrialised working-age populations are affected by clinical mental disorders: with point-prevalence estimates of 5% for severe mental disorders and another 15% for moderate mental disorders [1]. Among those affected, those with common mental disorders - depression, simple phobia, and generalised anxiety disorder - have the highest workforce participation rates [3]. In Australia, for example, the 2007 National Survey of Mental Health and Wellbeing estimated that 15% of the working population had a history of major depressive disorder (lifetime prevalence [7]); of these:

• 21% reported depressive symptoms in the past year and were in treatment

• 17% reported depressive symptoms in the past year and were not in treatment

• 11% were recovered and in treatment

• 52% were recovered and not in treatment.

In addition to clinical disorders, subclinical mental health problems and generalised distress are also prevalent in the working population [8]. In summary, mental health disorders and related problems represent a large and complex phenomenon in the workplace.

Mental health problems among working people are also costly to society at large, healthcare systems, employers, and affected individuals and their families. Conservative estimates of economic costs for European Union countries are 3-4% of gross domestic product [1,9]. Social costs include rising disability rates across the OECD due to mental disorders [1]. Healthcare costs for mental disorders vary widely, corresponding roughly with varying severity. For example, an Australian costing study found the greatest costs of depression amongst working people were borne by employers (far exceeding healthcare costs), with turnover costs figuring more prominently than presenteeism and absenteeism costs [7]. Costing studies to date, however, are limited in their ability to quantify costs to affected individuals and their families, particularly in regard to important social costs related to workplace stigma and discrimination [7].

### Premise Two: working conditions are an important modifiable risk factor for mental health problems

A substantial body of research has demonstrated the links between psychosocial working conditions—or job stressors—and worker health over the last three decades. Karasek and Theorell’s demand-control model has been particularly influential [10]. This model hypothesises that high job strain, defined by a combination of low control over how the job is done in the face of high job demands, will be harmful to health. This was first demonstrated in relation to cardiovascular disease outcomes [10,11]. Subsequent studies have found that job strain also predicts elevated risks of common mental disorders, even after accounting for other known risk factors [12-14]. While there is a considerable body of evidence supporting a dominant 'normal causation' model regarding the impact of working conditions on employee mental health, it should be noted that reversed causality, that is the impact of mental health on the assessment of working conditions can also occur. There is some evidence that working conditions and mental health influence each other reciprocally and longitudinally [15]. Systems thinking suggests bi-directional non-linear relationships [16] and better understanding of these processes using advanced analytic techniques (e.g., marginal structural modelling) and stronger study designs will undoubtedly be the subject of continuing research.

Numerous other job stressors, either individually or in combination, have been shown to influence mental health [14,17,18]. These include job insecurity, bullying or psychological harassment, low social support at work, organisational injustice, and effort-reward imbalance [12,14]. Unlike many historically prominent occupational exposures (e.g., asbestos), to which only a small proportion of the working population were exposed, *all working people* can be potentially exposed to job stressors. This means that even small increases in risk from such exposures can translate to substantial—and preventable—illness burdens. Given the population prevalence of a given exposure and the associated increase in risk for a specific outcome, the proportion of that outcome attributable to the exposure of interest can be estimated [19]. Based on job strain prevalence estimates of 18.6% in males and 25.5% in females and an odds ratio of 1.82 for job strain and depression [12], this method yielded estimates of job strain-attributable risk for depression in an Australian working population sample as 13% of prevalent depression among working males and 17% among working women [20]. More recently, comparable estimates were obtained from a study of the French working population for job strain-attributable risk for common mental disorders: 10.2–31.1% for men, 5.3–33.6% for women. Using a different approach, a New Zealand birth cohort study estimated that, at age 32, 45% of incident cases of depression and anxiety in previously healthy young workers were attributable to job stress [21]. While further research is needed to firmly establish the causality and magnitude of association of job strain and other stressor exposures in relation to common mental health problems (which would suggest that the attributable risks just presented are over-estimates), such single-exposure single-outcome estimates may also underestimate the proportion of mental health disorders attributable to job stressors, as a comprehensive estimate would account for all relevant job stressors and the full range of associated mental health outcomes [7]. In addition to depression, exposure to various job stressors has been associated with burnout, anxiety disorders, alcohol dependence, suicide and other mental health outcomes [14,22]. As such, preventing or reducing exposure to job stressors and improving the psychosocial quality of work could prevent a substantial proportion of common mental health problems. Such improvements would benefit other health domains as well, as exposure to these same job stressors also predicts elevated risks for poor health behaviours as well as other high burden chronic illnesses, including cardiovascular disease [23,24].

## Discussion

What then is the potential for preventing and managing this large and complex burden of mental health problems in the working population? The identification of modifiable risk factors implies potential preventability, but this needs to be demonstrated through intervention studies. Intervention strategies should be based on sound principles or theory, and their feasibility and effectiveness need to be demonstrated in implementation and effectiveness studies [25]. Below we summarise evidence in this regard for the three threads of our proposed integrated intervention approach to workplace mental health.

### Thread 1: protect mental health by reducing work–related risk factors

The relevant intervention principles and evidence in this area come predominantly from the fields of public health (e.g., occupational health and safety, health promotion) and psychology (particularly organisational psychology). Like other public health interventions, job stress prevention and control interventions can be directed at the primary, secondary, or tertiary levels [26-29]. Primary intervention aims to prevent the incidence of work-related mental health problems; it is ‘work-directed’ - aiming to reduce job stressors at their source by modifying the job or the work environment. Secondary intervention is *ameliorative* and ‘worker-directed’; it aims to modify how individuals respond to job stressors, usually through strategies to improve employees’ ability to cope with or withstand stressors. Secondary level intervention can also prevent the progress of sub-clinical mental health problems to diagnosable disorders. Tertiary intervention is *reactive* in that it responds to the occurrence of mental health problems; it involves treating affected workers and supporting rehabilitation and return-to-work. Theoretically, tertiary (and to some extent secondary) intervention can reduce the burden of mental disorders through early detection and treatment and limiting severity or chronicity. Some intervention strategies can be classified in different ways (e.g., increasing worker resilience or coping capacity could be considered primary prevention if it is done before a mental health problem has occurred, and secondary if it prevents the progression of an existing one)—most importantly, primary, secondary, and tertiary intervention are complementary, thus a comprehensive or systems approach to prevent and control the impacts of job stress entails all three [26]. In the preventive medicine typology (as relevant to thread 3 below), this framework roughly parallels universal, selected and indicated disease prevention [30].

Systematic reviews of job stress prevention and control studies show that the most effective interventions combine primary prevention to reduce job stressors with secondary intervention to strengthen workers’ abilities to withstand stressors [4,31-34]. While these systematic reviews indicate *what to do*, the more challenging question in application to policy and practice is *how to do it*. While the principles of intervention are broadly applicable, solutions are unique to the work context (e.g., worker socio-demographics and occupational skill levels, type of workplace, presence or absence of a union). For example, strategies to improve job control for a sales clerk will differ from strategies to achieve the same for a manager, even in the same workplace. Intervention design and implementation capabilities and resources in small-medium business settings also need to be considered [35]. Intervention strategies need to be tailored and context appropriate [28,36], making the development of such interventions more involved and labour-intensive than interventions for most other occupational hazards (e.g., installing a machine guard to prevent hand injuries).

Whilst knowledge of solutions for various work contexts is growing, there is still a need to apply principles and develop solutions on a case-by-case basis. This has likely contributed to the slow uptake of effective job stress prevention and control strategies in practice. Further, there is a persisting disconnect between evidence-based best practice and what is currently being undertaken in the workplace setting to address mental health, with prevalent practice directed more at secondary than primary intervention. For example, when Human Resources or OH&S staff are asked about their organisation’s response to job stress concerns, the most common response is to provide an Employee Assistance Program [37,38]. Other barriers to the uptake of evidence-based best practice include issues of stigma similar to those concerning mental illness in general, such as a persisting view of job stress as an individual weakness [38].

To summarise, job stress prevention and control is distinguished by its emphasis on primary or universal prevention, and the need to intervene at the level of work organisation as well as the individual. Implementation in practice, however, has proven challenging, in part because solutions need to be context-specific.

### Thread 2: promote mental health by developing the positive aspects of work as well as worker strengths and positive capacities

The relevant intervention principles and evidence in this area come predominantly from the field of psychology, in particular the rapidly developing field of positive psychology [39]. Positive psychology is defined as the study of “the conditions and processes that contribute to the flourishing or optimal functioning of people, groups, and institutions” [40]. What distinguishes positive psychology intervention in practice is that it applies strength-based methods to the achievement of positive outcomes. Strength-based methods aim to identify and enhance strengths or what is being done well, rather than trying to identify and fix what is ‘wrong’ in an individual, group or organisation [41]. It includes the application of methods such as appreciative inquiry, which involves asking positive questions in order to strengthen positive potential and create change, future search, which involves working towards an aspirational view of the future, and future inquiry—a hybrid of the two that acknowledges the views of all relevant stakeholders, generates respect for what has been done well, identifies a shared aspirational view of the future, and plans steps to move in that direction [42,43]. Positive outcomes include subjective wellbeing, psychological capital, positive mental health, employee engagement, and positive organisational attributes such as authentic leadership, supportive workplace culture and workplace social capital. Wellbeing—also referred to as subjective or psychological wellbeing, happiness or life satisfaction—is more than the absence of ill-health states but the presence of positive feelings and functioning [44]. The concept has also been applied to the domain of work [45]. A key point here is that the term ‘well-being’ does not refer to the absence of the negative; instead, wellbeing is most correctly defined and measured as the presence of positive feelings and functioning. Despite this important distinction, some inappropriately use ‘mental health and wellbeing’ as a catchall phrase for mental (ill) health constructs.

There is a need for both organization-wide and individual level approaches to employee well-being and mental health. This would align with the comprehensive or systems approach to job stress prevention described above. Importantly, positive approaches aim to promote the positive aspects of work and worker capabilities (including wellbeing) as distinct from other strategies, which aim to increase understanding of, or prevent, mental illness (e.g., mental health promotion and stress prevention). Some key approaches involve developing positive workplaces by establishing positive leadership practices, ensuring work is meaningful, and building a positive organizational climate [46,47]. The newness of positive approaches is reflected in its being the least commonly applied in organisational practice compared to the other two threads of our proposed integrated approach [48]. Positive psychology interventions, however, are becoming increasingly popular in clinical and general settings.

A meta-analysis of the general literature (in all settings) concluded that wellbeing can be sustainably enhanced and depressive symptoms reduced through positive interventions [49]. Positive-focused workplace strategies are less commonplace and need further development. This is particularly critical given the lack of intervention effectiveness in the workplace mental health space generally. Nevertheless, there are some small but successful examples in workplace settings, such as a positive psychology-based employee wellbeing program in a sample of working adults that showed positive changes in wellbeing over six months in comparison to non-participants [50]. Whilst research on strength-based methods, and particularly how to apply these methods at a primary level, is relatively new, research in this area is growing rapidly and may provide a valuable complement to problem-based methods.

The promise of positive approaches is clearly supported by established knowledge of the substantial positive influences of good quality work on mental health and wellbeing. In addition to the income and socio-economic position that paid work can provide, it can also positively impact adult socialisation, the development of identity, and the building of social connections extending beyond family and neighbourhood groups [14,51]. Furthermore, work can provide purpose and meaning, thus enhancing both self-efficacy and self-esteem, both of which protect and promote mental health. For example, research into what motivates older workers to stay in the labour market has demonstrated that opportunities to use their skills, to be creative, to gain a sense of accomplishment, and opportunities to interact with co-workers, are often rated more highly than financial security in decisions about staying in the labour market [52-54]. As well as having direct relevance to developing strategies to promote the positives of work for mental wellbeing, such findings are directly relevant to developing policy and practice responses to the ageing workforce across the industrialised world. This highlights the need for positive approaches to address eudaimonic (meaning and purpose) as well as hedonic (positive emotional, or happiness) aspects of workplace wellbeing [14,44].

To summarise, positive approaches provide a valuable and but rarely utilised complement to risk-based or negatively framed approaches (such as OH&S). However interventions involving positive work psychology are limited by their emphasis to date on the individual level [40] and the need for further evidence of effectiveness. Team/group and organisational level positive approaches are being developed, and may prove to yield greater benefits than individual-level approaches in the future.

### Thread 3: address mental health problems among working people regardless of cause

Work in this area has expanded rapidly over the last decade and has been largely developed from an illness or medical perspective, emphasising tertiary and secondary-level interventions. Workplace programs that aim to address mental health problems or disorders in the workplace commonly use psychoeducation and aim to improve mental health literacy, or develop skills for early intervention and the promotion of help-seeking [55,56]. An example of a program being implemented in multiple OECD countries is *Mental Health First Aid* (MHFA), which seeks to improve mental health literacy by developing knowledge and skills on how to recognise common mental disorders and provide “First Aid” support until professional help can be obtained, increasing understanding about the causes of mental disorders, improving knowledge of the most effective treatments, and reducing stigma [55,57]. There is evidence of effectiveness of MHFA from various studies [57] including two randomised-controlled trials conducted in workplace settings [55,58]. In addition to improvements in mental health literacy, there is also some evidence of improvements in mental health among MHFA trainees [55]. Further, there is evidence for the effectiveness of secondary and tertiary approaches to workplace suicide prevention in specific at-risk occupations such the U.S. air force [59]. Nevertheless, additional intervention studies as well as evidence synthesis is clearly warranted, and adequate numbers of specific types of intervention studies (e.g., workplace mental health literacy) may soon be available to enable systematic review and meta-analyses.

Other strategies for addressing mental health problems in the workplace focus on organisational culture and attitudes in relation to mental illness stigma and norms around disclosure. Mental health stigma in workplaces is a pervasive challenge, just as it is in broader society [60]. A study of 6,399 employees from 13 workplaces in the USA found that although 62% knew how to access company resources for depression care, only 29% indicated they would feel comfortable discussing the issue with their supervisor [61]. Unsupportive organisational culture and norms around depression disclosure are a contributing factor. Managers’ and leaders’ attitudes play a central role in changing these norms and are a priority target for intervention [62,63]. The development and dissemination of accommodation strategies is also needed, as managers , HR professionals, and others in workplaces may be willing but unsure about how to accommodate a worker with a mental health condition (compared to knowledge about physical accommodation), or these accommodations may be seen as too complicated to put in place [64-66]. Finally, some strategies focus on the role of organisational culture in improving return-to-work from a mental illness-related absence [67].

To summarise, illness-focused approaches to addressing mental health problems are strongest at the tertiary and secondary, or—in preventive medicine terminology—selected and indicated levels. Initially, they tended to be individual-focused, but strategies are rapidly expanding to address organisational culture and norms. There is promising evidence of effectiveness, but further research in this regard is needed. Early detection and disclosure are hampered by persisting stigma and the potential for discrimination; the continuing improvement of strategies to address these barriers is a key priority for research, policy, and practice.

### The integrated approach: joining the threads

A defining feature of the integrated approach is the mutually reinforcing nature of the three threads. While the protective focus of the first thread aims to identify and address factors that can undermine the mental health of employees – and therefore encourages employers to fulfil their responsibility to provide a safe and healthy working environment, the overall goal of the second thread is to complement the risk reduction approach by promoting those characteristics that can strengthen individual and organisational health and can lead to high levels of positive wellbeing. To some extent this complementarity is already apparent; for example, understanding of the importance of job control has evolved from two sides of the same coin. Low job control was identified in public health research as an important risk factor for mental health problems (thread 1), and the promotion of autonomy (or high job control) is a common strategy in positive approaches (thread 2). Maintaining this dual protection-promotion emphasis can benefit workplace mental health in many ways, not least in encouraging organisations and their representatives to examine the strengths and weaknesses of their working environments, to keep a more ‘balanced scorecard’ in relation to monitoring the performance of their various systems, policies and practices, and to properly identify and mobilise the resources available in their organisations to build workplaces that are not just safer and fairer but are also more attractive to and engaging for employees.

The third thread can complement the first two in various ways. Certain knowledge and awareness aspects of mental health literacy (MHL), for example, relate directly to the other two threads. The workplace MHL strategies we have piloted for example, highlight that poor working conditions and job stress are modifiable risk factors for common mental health problems, and (where applicable) that there are legislative OH&S mandates to protect psychological as well as physical health [68,69], thus building employee awareness of and employer commitment to the need to address working conditions (linking to thread 1). Workplace MHL can also highlight the protective value of resilience in relation to mental disorders, building motivation for and commitment to positive approaches (linking to thread 2). In addition, starting where organisations are receptive (MHL training) can provide the encouragement/incentives to employers (near term improvement in MHL) needed to sustain employer interest and commitment to the improvement of working conditions and job quality over the longer term. This could help provide entrée into workplaces that might not otherwise consider job stress or other mental health interventions on their own, increasing the reach and uptake of the full integrated approach.

The growing public awareness and employer receptivity to MHL intervention suggests that the integrated approach might best be described as *workplace mental health literacy*. Based on Jorm’s earlier definition of MHL as “knowledge and beliefs about mental disorders which aid their recognition, management or prevention” [70], we would define workplace mental health literacy the knowledge, beliefs, and skills that aid in the prevention of mental disorders in the workplace, and the recognition, treatment, rehabilitation, and return to work of working people affected by mental disorders. Differing, but overlapping sets of knowledge, beliefs and skills would apply to people in various roles in or in the relation to the workplace setting, including for examples workers, managers, and HR staff in a given workplace, and worker and employer advocates and healthcare professionals in relation to various workplaces.

Further work will be required to articulate the links and genuinely integrate the threads of the integrated approach, which may indeed lead to efficiencies in implementation as well as preventive synergies, such as has been realised through integrated approaches targeting cancer prevention other aspects of workplace health [71-73].

### The integrated approach: cautionary notes

Although combining the three threads of the integrated approach could substantially improve mental health outcomes over above what might be achieved by each thread on its own, it is important to acknowledge the potential risks and challenges of adopting this approach. To date, there is a persisting over-emphasis on individual-directed intervention in workplace health intervention policy and practice, which would need to be overcome in order to realise a genuinely integrated approach. The great uptake of workplace mental health literacy as well as resilience-oriented positive psychology programs may be partly explained by this. For example, past mental health literacy programs have been largely individual-directed education and training programs, thus far mainly evaluated in terms of short-term changes in individuals’ knowledge, attitudes, and helping skills. In contrast, reducing job stressors and improving job quality requires organizational changes, which generally require more resources and a longer period of change. In a recent feasibility study to develop and implement an integrated job stress and mental health literacy intervention, significant improvements in mental health literacy were observed over one year, but—disappointingly—no improvements in job demands, job control, or workplace social support [68,69]. More intensive or sustained work-directed intervention, longer follow-up, or both are needed to achieve and demonstrate improvement in working conditions.

There is also a risk with integrated approaches of employers confusing mandatory and voluntary responsibilities. In Australia and many other OECD countries, there is legal obligation to provide psychologically as well as physically safe working condition under OH&S law. Yet, employers seem to embrace workplace mental health literacy and related programs more readily than job stress prevention. Unions and other worker advocates are understandably concerned that employer responses to mandatory requirements might be confused with or diluted by responses to voluntary programs. There is a need for improved articulation of all legal and ethical requirements, including employment, anti-discrimination, and equal opportunity as well as OH&S law, relevant to workplace mental health, as a component of integrated approaches, for the benefit of employers, workers, and other workplace stakeholders. The protection of confidentiality and the prevention of discrimination are also key considerations in integrated and other workplace mental health interventions.

Finally, to realise the greatest possible population mental health benefits, governments and other policy-makers will need to consider how to ensure interventions are accessible to those workers who are most in need of them. Lower occupational status workers have the highest prevalence of mental health problems, the greatest exposure to job stressors, and the lowest quality jobs [27,74]. These groups are typically the least likely to receive job stress or other workplace mental health intervention. In Australia and some other OECD countries, exceptions include blue-collar males who have been prioritised for workplace mental health literacy intervention by governments and non-governmental organisations, such as mental health promotion foundations. This is largely on the basis of their low help-seeking behaviours and high prevalence of mental health problems. This praiseworthy policy action could be further strengthened by the integration of interventions that reduce job stressors and improve work quality [74]. In the absence of concerted efforts to reach priority groups, population level implementation of integrated or other workplace mental health intervention risks the exacerbation of mental health inequalities, as more advantaged groups would be more likely to experience and benefit from intervention than disadvantaged groups, resulting in widening disparities similar to those seen from population-level tobacco control and other health promotion interventions [75,76].

### Next steps for developing the integrated approach

There are various hopeful signs for the development of integrated approaches in practice, policy, and research. There is growing receptivity among employers and other workplace stakeholders to the value of integrated approaches, stemming largely from growing awareness of the widespread prevalence and the impact of mental health problems (work-related or otherwise) on productivity at work (e.g., sickness absence, presenteeism) [3,7,35], as well as from growing recognition of the need to fulfill OH&S obligations with respect to the protection of psychological as well as physical health.

Integrated approaches are also developing to some extent in policy and practice across the OECD. In addition to the example previously discussed, Canada very recently published the first *Standard for Psychological Health and Safety in the Workplace* in 2013 [77], the European Agency for Safety and Health at Work published *Mental Health Promotion in the Workplace* in 2011 [78], the WHO has published generic guidance on integrated approaches (for workplace health in general) [79] as well as specific workplace suicide prevention guidance [80].

While these policy and practice developments are very encouraging, there is a dearth of effectiveness evaluation studies on these programs and intervention guidance resources. Intervention research on these and other integrated approaches should be a high priority. This would include the full spectrum of intervention research: development, implementation, and effectiveness [25]. Developmental research (developing *what to do* and *how*) is a particular priority for positive approaches, as most of the above examples focus little or not at all on how to promote the positive aspects of work. As each intervention approach has evolved relatively independently, there is a need for further improvement in the integration of strategy and guidance material from the three threads; this would best be achieved through the involvement of the full range of workplace stakeholders. For example, we have recently applied the Delphi consensus method to work with three stakeholder groups (managers, workers, and workplace health professionals) to develop [81] and web-publish (http://www.prevention.workplace-mentalhealth.net.au) a set of integrated guidelines for the prevention of mental health problems in the workplace, extending similar practice-based developmental research [67] to produce guidelines for return to work from a mental illness (http://returntowork.workplace-mentalhealth.net.au/). The recent Canadian standard for Psychological H&S in the Workplace is another source of guidance on integrated approaches to workplace mental health [77].

Implementation research is also needed to inform both policy and practice (e.g., to answer research questions such as: What factors facilitate or hinder implementation? What levels of support do various types and sizes of organisations need to implement integrated approaches? What is practically achievable for organisations implementing their own programs?). Finally, effectiveness studies are needed to demonstrate that integrated approaches work (e.g., When implemented as intended [82], are there significant improvements in mental health literacy, working conditions, and job quality over time? When implemented as intended, are there improvements in mental health and wellbeing over time?). Economic studies (cost-effectiveness, cost-benefit) will also be required alongside effectiveness studies to make the business case. While the costing studies described under Premise 1 above show that there are potential savings to be made, health economic evaluation research to date on worksite mental health interventions is limited. A recent meta-analysis of 10 studies in this area found that they covered mainly screening and return-to-work interventions in isolation, and found limited evidence of positive cost-benefit ratios for screening and treatment interventions and no favourable cost-effectiveness for return to work interventions [83]. It remains to be seen whether integrated approaches would yield better results.

To optimise the translation of research to practice, the applied intervention research described above would be conducted in partnership with organisations and workplace stakeholders by multi-disciplinary teams of researchers (at least covering disciplines relevant to each of the three threads of the integrated approach). This will involve engagement and collaboration by researchers with relevant decision-makers and other workplace stakeholders [1], and represents a move towards viewing practice-based evidence as equally relevant as evidence-based practice [84].

### Linking interventions in the workplace with other settings

Whilst this paper is specifically focused on intervention in the workplace setting, we acknowledge that workplaces also interface with other important settings and contexts for mental health intervention in the working population. Most proximal to the workplace, there are those workers who have left work temporarily on sickness absence or workers’ compensation claims due to a mental health problem, and who need to return to work with the same employer. This may involve return to work from a mental health problem that is work-related, not work-related, or some combination of the two. This is an area of active research, policy, and practice development. While research in this area is still evolving, there is a growing recognition that the strategies to return workers with mental health problems to the workplace are likely different from those commonly used to accommodate workers with physical conditions [64-66,85,86]. In addition, workers with mental health conditions may be more susceptible to recurrent episodes of absence [87,88]. A recent study of workers with a previous sickness absence due to mental health conditions in the Netherlands identified that workers who had conflicts with their supervisor were more likely have a recurrent absence [89]. These same researchers have also demonstrated that a problem solving intervention, focused on processes to identify and address challenges in staying at work (including consultations between the work and the supervisor) was effective in reducing the likelihood of recurrent sickness absences compared to care as usual [90]. While more research is needed to determine if these results are generalizable to other countries or settings (or if this type of interventions is feasible in other settings) this finding supports the notion that aspects of the workplace play an important role in reducing sickness absence due to mental health conditions, and in facilitating successful return to work mental health-related absence.

There is ample evidence that job loss is associated with a decline in mental health [91,92]. The point of departure from the employer (e.g., with redundancy, downsizing, restructures—events which appear to be increasing in frequency [93]) represents one opportunity for intervention. While many employers offer job seeking support or job retraining, and it can also be valuable to acknowledge potential mental health impacts and to encourage help seeking in the event that it becomes needed. An Australian mental health foundation, *beyondblue*, has established a resource entitled “Taking Care of Yourself After Retrenchment or Financial Loss” for use in such circumstances by employers and others (available at http://www.beyondblue.org.au). Once separated from an employer and established as unemployed, social welfare, trade union, NGO, or other stakeholders can offer further assistance towards re-employment as well as mental health literacy and help-seeking education. Some such programs in the US and Finland have shown evidence of prevention of job loss-related declines in mental health as well as improved re-employment outcomes [94-96]. Further development of such programs is warranted to address mental health declines and increased suicide risks associated with unemployment [97,98].

## Conclusions

An integrated approach to workplace mental health can expect near-term improvements in mental health literacy, to be followed by longer-term improvements in working conditions and job quality—given adequate organizational commitment, support, and time to achieve organizational change. These changes should, in turn, lead to improvements in mental health and wellbeing. While improvements in psychosocial and other working conditions may be more difficult to achieve than improvements in mental health literacy, we would argue that efforts should continue to be made in this regard in order to fulfil legal and ethical mandates to provide psychologically safe work and to reduce the substantial burden of work-related mental health problems. Increasing awareness of work-related influences on mental health, and the growing recognition of the need for ‘psychologically safe’ work may help to drive organisational efforts to improve psychosocial working conditions.

Developing an integrated approach could also be framed as moving towards a comprehensive notion of *workplace mental health literacy* as involving the knowledge, beliefs, and skills that aid in the prevention of mental illness in the workplace, and the recognition, treatment, rehabilitation, and return to work of working people affected by mental illness. This includes consideration of working conditions and their influence on mental health (positive as well as negative), as well as addressing mental health problems among working people regardless of cause.

## Abbreviations

MHFA: Mental Health First Aid; MHL: Mental health literacy; OECD: Organisation for Economic Cooperation & Development; OH&S: Occupational Health & Safety.

## Competing interests

The authors declare that they have no competing interests.

## Authors’ contributions

All authors contributed to the conception and structure of the manuscript. LaMontagne, Martin, and Page drafted the paper, and all authors contributed to revisions, reviewed and approved the final manuscript.

## Pre-publication history

The pre-publication history for this paper can be accessed here:

http://www.biomedcentral.com/1471-244X/14/131/prepub
